# Genomic organization of repetitive DNAs and its implications for male karyotype and the neo-Y chromosome differentiation in *Erythrinus erythrinus* (Characiformes, Erythrinidae)

**DOI:** 10.3897/CompCytogen.v8i2.7597

**Published:** 2014-07-14

**Authors:** Cassia Fernanda Yano, Luiz Antonio Carlos Bertollo, Wagner Franco Molina, Thomas Liehr, Marcelo de Bello Cioffi

**Affiliations:** 1 Departamento de Genética e Evolução, Universidade Federal de São Carlos, Rodovia Washington Luís (SP 310) Km 235, São Carlos, SP, Brazil; 2 Departamento de Biologia Celular e Genética, Centro de Biociências, Universidade Federal do Rio Grande do Norte, Natal, RN, Brazil; 3 Jena University Hospital, Friedrich Schiller University, Institute of Human Genetics, Kollegiengasse 10, D-07743 Jena, Germany; 4 Professor Sênior at Universidade Federal de São Carlos

**Keywords:** FISH, microsatellites, retrotransposable sequences, sex chromosomes

## Abstract

Studies have demonstrated the effective participation of repetitive DNA sequences in the origin and differentiation of the sex chromosomes in some biological groups. In this study several microsatellites and retrotranposable sequences were cytogenetically mapped in the *Erythrinus erythrinus* (Bloch & Schneider, 1801) male genome (karyomorph C), focusing on the distribution of these sequences in the sex chromosomes and in the evolutionary processes related to their differentiation. Males of *E. erythrinus* – karyomorph C – present 2n = 51 chromosomes (7m + 2sm + 6st + 36a), including the X_1_X_2_Y sex chromosomes. The C-positive heterochromatin has a predominant localization on the centromeric region of most chromosome pairs, but also in some telomeric regions. The 5S rDNA sites are located in the centromeric region of 27 chromosomes, including 26 acrocentric ones and the metacentric Y chromosome. The retrotransposons *Rex* 1 and *Rex* 6 show a dispersed pattern in the karyotype, contrasting with the *Rex* 3 distribution which is clearly co-localized with all the 27 5S rDNA sites. The microsatellite sequences show a differential distribution, some of them restricted to telomeric and/or interstitial regions and others with a scattered distribution on the chromosomes. However, no preferential accumulation of these elements were observed in the neo-Y chromosome, in contrast to what usually occurs in simple sex chromosome systems.

## Introduction

Sex chromosomes have been widely studied in several invertebrate, vertebrate and plant individuals, focusing on their origin and differentiation (Kubat et al. 2008, Ezaz et al. 2009, Kaiser and Bachtrog 2010, Cioffi et al. 2011, 2013), providing excellent opportunities to investigate the evolutionary processes acting on the genome (Bachtrog et al. 2011). Regarding fishes, different sex chromosome systems can occur, from simple to multiple ones (Devlin and Nagahama 2002), in which repetitive DNA sequences have been increasingly used in order to investigate the evolutionary processes of sex chromosome differentiation (Koga et al. 2002, Lippman et al. 2004, Gross et al. 2009, Cioffi et al. 2011, Martins et al. 2012). In fact, repetitive sequences can accumulate in the sex-specific chromosome due to the reduction of the recombination rate between the proto-sex pair, thus contributing to its differentiation (Vallender and Lahn 2004, Charlesworth et al. 2005).

Repetitive sequences include different classes of *in tandem* repeats, such as satellite DNAs, minisatellites and microsatellites, and interspersed repeats, like the transposable elements (TEs) (Jurka et al. 2005). Microsatellites are constituted by short sequences from 1 to 6 base pairs and, as such, classified as mono-, di-, tri-, tetra-, penta and hexanucleotides (Schlötterer and Harr 2001). Concerning the TEs, they can be grouped into two categories: the retrotransposons, which move into the genome via an intermediate RNA, and the transposons, which are directly transposed into the genome through a DNA copy (Charlesworth et al. 1994, Kazazian 2004).

Erythrinidae (Characiformes), are a small Neotropical fish family composed of three genera, *Erythrinus* Scopoli, 1777, *Hoplerythrinus* Gill, 1896 and *Hoplias* Gill, 1903 (Oyakawa 2003). Among the species of this group, *Hoplias malabaricus* (Bloch, 1794) and *Erythrinus erythrinus* (Bloch & Schneider, 1801) present a great diversity of karyomorphs and differentiated sex chromosome systems (Bertollo et al. 2000, Bertollo et al. 2004). In fact, four karyomorphs (A to D) were already described for *Erythrinus erythrinus*, and with exception of karyomorph A that not have differentiated sex chromosomes, the karyomorphs B, C and D share an X_1_X_1_X_2_X_2_/X_1_X_2_Y multiple sex system, but with different diploid numbers and chromosome morphology (Bertollo et al. 2004).

In this study several microsatellites and retrotransposable sequences were cytogenetically mapped in the *Erythrinus erythrinus* male genome (karyomorph C), focusing on their distribution in the sex chromosomes and in the evolutionary processes related to the differentiation of the neo-Y chromosome.

## Methods

### Material collection and classical cytogenetic analyses

Six male specimens of *Erythrinus erythrinus* (karyomorph C), from the Manaus region (3°13'41.4"S, 59°43'43.1"W – Amazon State, Brazil) were analyzed. The experiments followed ethical conducts, and anesthesia was used prior to sacrificing the animals. Mitotic chromosomes were obtained from the anterior portion of the kidney, according to Bertollo et al. (1978). In addition to the standard Giemsa staining, the C-banding method (Sumner 1972), was also employed to detect the distribution of the C-positive heterochromatin on the chromosomes.

### Probe preparation

Oligonucleotide probes containing microsatellite sequences (CA)_15_, (CAA)_10_, (CAC)_10_, (CAG)_10_, (CAT)_10_, (CGG)_10_, (GA)_15_, (GAA)_10_, (GAG)_10_ and (TA)_15_ were directly labeled with Cy5 during synthesis by Sigma (St. Louis, MO, USA), as described by Kubat et al. (2008). The retrotransposable elements *Rex* 1, *Rex* 3 and *Rex* 6 were obtained by PCR according to Volff et al. (1999). The 5S rDNA probe included 120 base pairs (bp) of the 5S rRNA encoding gene and 200 bp of the non-transcribed spacer (NTS) (Martins et al. 2006). All these probes were labeled with DIG-11-dUTP using DIG-Nick-translation Mix (Roche), and used for the fluorescence *in situ* hybridization (FISH) experiments.

### Fluorescence *in situ* hybridization and signal detection

The FISH method was conducted as follows: slides with fixed chromosomes were maintained at 37 °C for 1 hour. Subsequently, they were incubated with RNAse (10 mg/ml) for 1 hour at 37 °C in a moist chamber. Next, it was performed a 5-minute wash with 1xPBS and 0.005% pepsin was applied to the slides (10 minutes at room temperature). The slides were then washed again with 1xPBS. The material was fixed with 1% formaldehyde at room temperature for 10 minutes. After further washing, the slides were dehydrated with 70%, 85% and 100% ethanol, 2 minutes in each bath. The chromosomal DNA was denatured in 70% formamide/2xSSC for 3 minutes at 72 °C. The slides were dehydrated again in a cold ethanol series (70%, 85% and 100%), 5 min each. The hybridization mixture, containing 100 ng of denatured probe, 10 mg/ml dextran sulfate, 2xSSC and 50% formamide (final volume of 30 μl) were heated to 95 °C for 10 minutes and then applied on the slides. Hybridization was performed for a period of 16-18 hours at 37 °C in a moist chamber. After hybridization, the slides were washed for 5 minutes with 2xSSC and then rinsed quickly in 1xPBS. The signal detection was performed using anti-digoxigenin rhodamine (Roche) for the 5S rDNA, *Rex* 1, *Rex* 3 and *Rex* 6 probes. Subsequently, the slides were dehydrated again in an ethanol series (70%, 85% and 100%), 2 minutes each. After the complete drying of the slides, the chromosomes were counterstained with DAPI/antifade (1.2 mg/ml, Vector Laboratories).

### Microscope analyses

Approximately 30 metaphase spreads were analyzed to confirm the diploid chromosome number, karyotype structure and FISH results. Images were captured by the CoolSNAP system software, Image Pro Plus, 4.1 (Media Cybernetics, Silver Spring, MD, USA), coupled to an Olympus BX50 microscope (Olympus Corporation, Ishikawa, Japan). The chromosomes were classified as metacentric (m), submetacentric (sm), subtelocentric (st) or acrocentic (a), according to their arm ratios (Levan et al. 1964).

## Results

**Figure 1.**
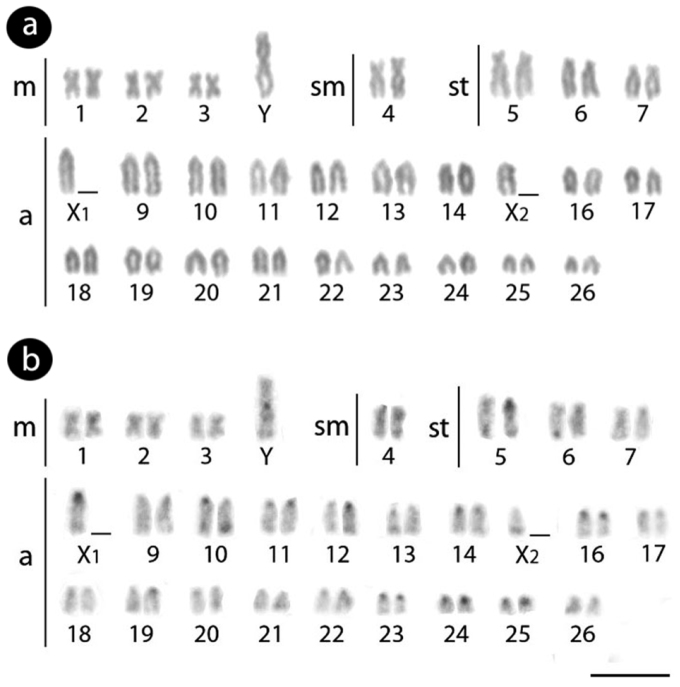
Male Karyotype of *Erythrinus erythrinus* arranged from Giemsa-stained (**a**) and C-banded chromosomes (**b**). Bar = 5 µm.

**Figure 2.**
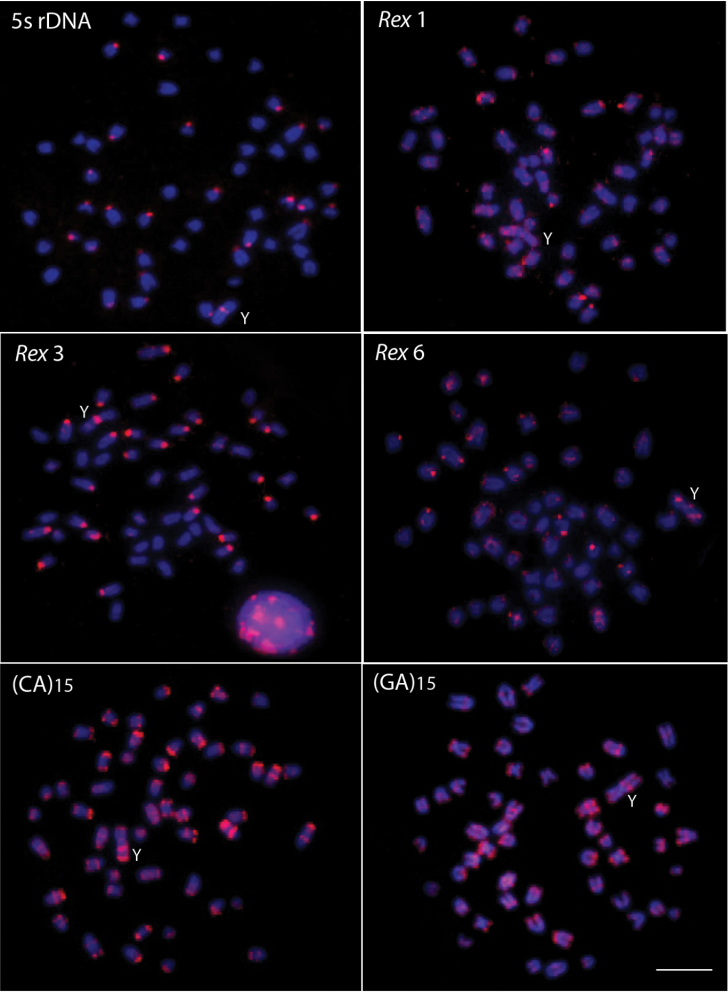
Male metaphase plates of *Erythrinus erythrinus* probed with 5S rDNA, *Rex* 1, *Rex* 3 and *Rex* 6 transposons and microsatellite sequences. Bar = 5 μm.

Males of *Erythrinus erythrinus* – karyomorph C – present 2n = 51 chromosomes (7m + 2sm + 6st + 36a), including the X_1_X_2_Y sex chromosomes. While the chromosomes X_1_ and X_2_ are acrocentric, the Y is the largest metacentric chromosome in the karyotype (Fig. 1a). The C-positive heterochromatin has a predominant localization on the centromeric region of most chromosome pairs, but also in some telomeric regions (Fig. 1b). The 5S rDNA sites are located in the centromeric region of 27 chromosomes, including 26 acrocentric ones and the metacentric Y-chromosome (Fig. 2). The retrotransposons *Rex* 1 and *Rex* 6 show a dispersed pattern in the karyotype, contrasting with the *Rex* 3 distribution which is clearly co-localized with all the 27 5S rDNA sites (Fig. 2).

**Figure 3.**
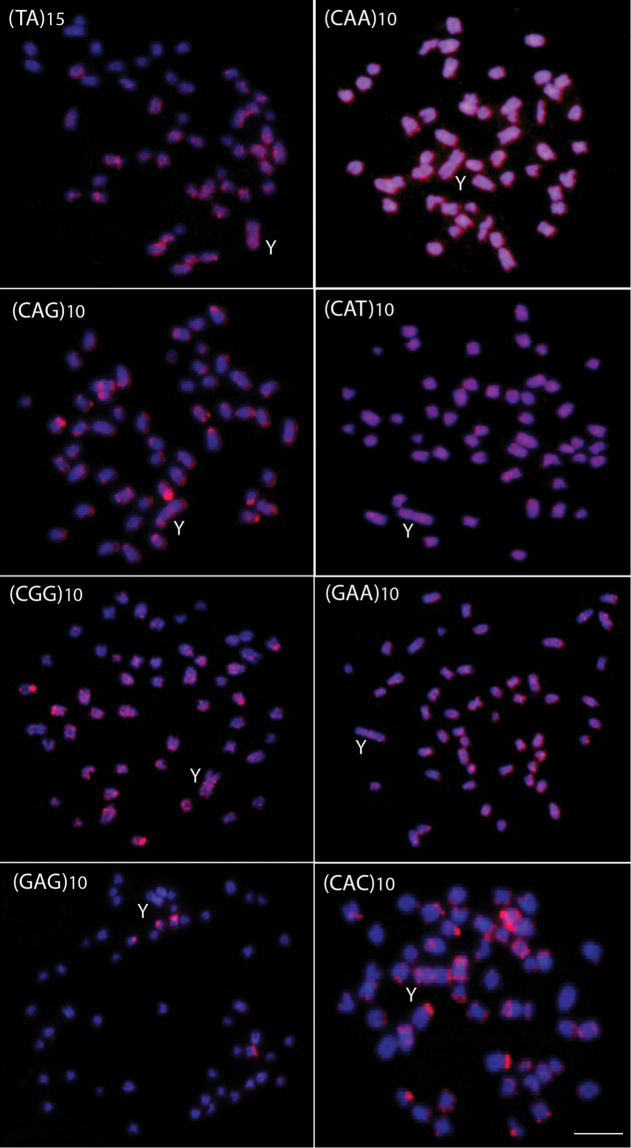
Male metaphase plates of *Erythrinus erythrinus* probed with microsatellite sequences. Bar = 5 μm.

**Figure 4.**
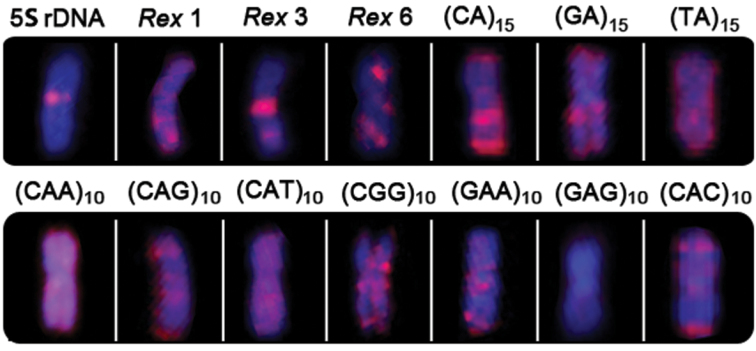
Distribution of repetitive DNA sequences in the Y chromosome of *Erythrinus erythrinus*.

The microsatellite sequences show a differential distribution, some of them restricted to telomeric and/or interstitial regions and others with a scattered distribution on the chromosomes. Microsatellites (CA)_15_, (GA)_15_, (CAC)_10_ and (CAG)_10_ are mainly accumulated in the telomeric regions of the chromosomes and in some interstitial sites, but with a different distribution, since some chromosomes present higher signals than other ones (Figs 2 and 3). However, the (CA)_15_ sequences present a greater distribution compared with the other three classes of microsatellites, including on the Y-chromosome. In fact, this chromosome show a greater accumulation for the (CA)_15_ microsatellite, mainly in interstitial and telomeric regions of the long arms. In turn, the microsatellites (CAA)_10_, (CAT)_10_, (CGG)_10_, (GAA)_10_ and (TA)_15_ present a dispersed distribution among the autosomes and on the Y chromosomes (Fig. 3). In contrast, (GAG)_10_ microsatellite is poorly represented in the genome of *Erythrinus erythrinus*, with only four chromosomes showing mapped sites in their centromeric region. The Y-chromosome shows no labeling for this microsatellite (Fig. 3). Figure 4 highlights the distribution of all repetitive sequences analyzed along the Y-chromosome of the species.

## Discussion

### General distribution of repetitive sequences in the whole genome

The repetitive fraction of the genome can be a useful tool for the identification of recent genomic changes that occurred during the evolutionary process. Retrotransposons usually carry regulatory sequences and may attract methylation, thus influencing the gene expression (Martin et al. 2009). In addition, these sequences can also be a substrate for chromosomal rearrangements, including inversions and translocations (Ozouf-Costaz et al. 2004).

The *Rex* family seems to be abundant in different teleost species (Gross et al. 2009), with a varied distribution, from a scattered pattern to a preferential accumulation in some regions of the chromosomes (Gross et al. 2009, Ferreira et al. 2011). *Rex* 3 has been the most analyzed retrotransposon in fishes, showing different distributional patterns in the genome of different species (Gross et al. 2009). In *Erythrinus erythrinus* *Rex* 3 showed a clear compartmentalized distribution in the centromeric region of the chromosomes, which was also observed in other fish species, such as *Notothenia coriiceps* Richardson, 1844 and *Chionodraco hamatus* (Lönnberg, 1905), with a compartmentalized distribution in the pericentromeric region (Ozouf-Costaz et al. 2004). As in the present study, scattered signals for the *Rex* 1 and *Rex* 6 retrotransposons were also found among the cichlid fishes, although many species also showed pericentromeric accumulation of these elements (Valente et al. 2011).

In *Erythrinus erythrinus* (karyomorph C), *Rex* 3 showed a clear colocalization with 5S rDNA sites in the centromeric region of several chromosomes. Our data agree with previous results achieved for this same karyomorph (Martins et al. 2012) and for karyomorph D (Cioffi et al. 2010), showing a surprising spreading of 5S rDNA/*Rex* 3 transposons in the genome of this fish, which contrasts with other karyomorphs of this species where the same event is not found. In this sense, in addition to classical cytogenetic rearrangements, these families of repetitive DNAs were useful to demonstrate the hidden biodiversity not detected by conventional morphological analyzes in this fish group. According to Volff et al. (1999), the *Rex* 3 retrotransposon can be associated with gene coding regions, as well as be inserted in introns and in the vicinity of promoter regions, thus probably allowing the dispersion of some genes with which they are associated. It is possible that such dispersion mediated by transposable elements is not a relatively rare event among fishes. In fact, a 5S rDNA dispersion was also recently found in the marine fish *Ctenogobius smaragdus* (Valenciennes, 1837), suggesting the mediation of repetitive elements (Lima-Filho et al. 2014). In addition, in *Rachycentron canadum* (Linnaeus, 1766) the Tol2 element, belonging to the family of hAT transposons, shows a huge colocalization with the 18S rDNA sites in the karyotype (Costa et al. 2013), indicating other TEs than those of the *Rex* family associated with ribosomal DNA families.

Microsatellites mapping has shown both similar as well as different distribution patterns between species (Kubat et al. 2008, Cioffi et al. 2010, Pokorná et al. 2011, Cioffi et al. 2012). This is also true for *Erythrinus erythrinus* where (CA)_15_, (GA)_15_, (CAC)_10_ and (CAG)_10_ microsatellites are mainly compartmentalized in the telomeric and interstitial regions of the chromosomes, while (TA)_15_, (CAA)_10_, (CAT)_10_, (CGG)_10_ and (GAA)_10_ microsatellites show a more scattered distribution throughout the genome. In turn, the (GAG)_10_ microsatellite is poorly represented in the genome of this species. Although (CA)_15_ and (GA)_15_ dinucleotides have a preferential accumulation in the telomeric regions of other fish species (Cioffi et al. 2011, Cioffi et al. 2012), they were also mapped in the interstitial region of several *Erythrinus erythrinus* chromosomes.

### Distribution of the repetitive sequences in the sex chromosomes

The cytogenetic mapping of repetitive DNAs has improved the knowledge of the evolutionary origin of the neo-Y chromosome. In fact, the chromosomal mapping of repetitive DNA sequences has shown differential accumulations on the sex-specific chromosomes (Kubat et al. 2008, Cioffi et al. 2012, Xu et al. 2013).

In *Erythrinus erythrinus*, a centric fusion was proposed to be related with the origin of the big metacentric Y chromosome found in karyomorphs B, C and D and the differentiation of the X_1_X_1_X_2_X_2_/X_1_X_2_Y multiple sex system in these karyomorphs (Bertollo et al. 2004). This proposal was strengthened by the colocalization of 5S rDNA/*Rex* 3 transposon in the centromeric region of several acrocentric chromosomes, and also of the metacentric Y-chromosome (Cioffi et al. 2010, Martins et al. 2012). Indeed, important role for DNA repetitive sequences, as the *Rex* family, has been assigned for chromosomal rearrangements and differentiation of the sex chromosome systems in fish species (Ozouf-Costaz et al. 2004, Cioffi and Bertollo 2012).

In turn, the mapping of microsatellites in the chromosomes has also been useful tools for analyzing the differentiation of sex chromosomes. In simple sex chromosomes, such as the ZZ/ZW system of *Leporinus reinhardti* Lütken, 1875 and *Triportheus auritus* (Valenciennes, 1850) (Cioffi et al. 2012), and the XX/XY system of *Hoplias malabaricus* – karyomorph B (Cioffi et al. 2010), there was a preferential accumulation of different microsatellites in the heterochromatic region of the sex-specific chromosome. However, in multiple sex chromosomes, such as the X_1_X_1_X_2_X_2_/X_1_X_2_Y system of *Hoplias malabaricus* – karyomorph D (Cioffi et al. 2011) and on the rock bream fish *Oplegnathus fasciatus* (Temminck & Schlegel, 1844) (Xu et al. 2013), although a preferential accumulation of some microsatellites was also found in the neo-Y chromosome, it was not so marked as in the simple sex chromosome systems.

Additionally, no preferential accumulation of microsatellites was found to occur in the sex chromosomes of *Erythrinus erythrinus*. In fact, there were no significant differences in the distribution of the microsatellites analyzed concerning to autosomes and sex chromosomes, based on the neo-Y chromosome which is easily identifiable in this species. It is well known that the suppression of the recombination is a crucial step in the differentiation of the sex pair, leading to the differentiation of the sex-specific chromosomes. Multiple sex chromosome systems originate from chromosomal rearrangements from simple systems and can itself reduce or eliminate the recombination near breakpoints, reinforcing previous suggestions that other events, such as accumulation of repetitive DNAs, may not be necessary for this process (Moreira-Filho et al. 1993, Vieira et al. 2003).

## Conclusion

The repetitive sequences used in this study did not show a differential accumulation in the neo-Y chromosome of *Erythrinus erythrinus*, showing a similar distribution to the other chromosomes of the complement. However, it is clear that different repetitive DNAs may exhibit differential distribution patterns in chromosomes, including the neo-Y one (Figure 4), probably reflecting differences in the time of chromosomal occupation, as well as of strategies for dispersal throughout the genome.
